# Preoperative prediction of postoperative cerebellar mutism syndrome. Validation of existing MRI models and proposal of the new Rotterdam pCMS prediction model

**DOI:** 10.1007/s00381-020-04535-4

**Published:** 2020-02-18

**Authors:** Dhaenens BAE, Van Veelen MLC, CE Catsman-Berrevoets

**Affiliations:** 1 Department of Pediatric Neurology, Erasmus Medical Center/ Sophia Children’s Hospital, Dr. Molewaterplein 40, 3015 GD Rotterdam, The Netherlands; 2 Department of Pediatric Neurosurgery, Erasmus Medical Center/Sophia Children’s Hospital, Dr Molewaterplein 40, 3015 GD Rotterdam, The Netherlands

**Keywords:** Postoperative cerebellar mutism syndrome, CMS, pCMS, Risk prediction, Brain tumor, Child

## Abstract

**Purpose:**

Postoperative cerebellar mutism syndrome (pCMS) is a complication that may occur after pediatric fossa posterior tumor surgery. Liu et al. developed an MRI-based prediction model to estimate pCMS risk preoperatively. The goal of this study was to validate the model of Liu et al. and if validation was not as sensitive in our group as previously described to develop an easy to use, reliable, and sensitive preoperative risk prediction model for pCMS.

**Methods:**

In this study, 121children with a fossa posterior tumor who underwent surgery at ErasmusMC/Sophia Children’s Hospital, the Netherlands between 2004 and 2018 could be included. Twenty-six percent of them developed pCMS. Preoperative MRI were scored using the Liu et al. model.

**Results:**

The Liu et al. model reached an accuracy of 78%, a sensitivity of 58%, and a specificity of 84% in our cohort. In a new risk model some of the variables of Liu et al. were included as well as some of the recently described preoperative MRI characteristics in pCMS patients by Zhang et al. The new model reached an accuracy of 87%, a sensitivity of 97%, and a specificity of 84% in our patient group.

**Conclusion:**

Because the Liu et al. model did not provide an as accurate risk prediction in our cohort as was expected, we created a new risk prediction model that reached high model accuracy in our cohort that could assist neurosurgeons in determining their surgical tactics and help prepare high risk patients and their parents for this severe complication.

**Electronic supplementary material:**

The online version of this article (10.1007/s00381-020-04535-4) contains supplementary material, which is available to authorized users.

## Introduction

Cerebellar mutism syndrome (CMS) may occur as a complication in up to 2–29% of children after posterior fossa tumor surgery [1, 2]. The core symptom of postoperative CMS (pCMS) is mutism or occasionally a very severe reduction of speech, which can be accompanied in varying combinations and severity by irritability, ataxia and hypotonia, long tract signs, cranial nerve palsies, oropharyngeal dyspraxia, and behavioral symptoms such as whining, high-pitched crying, and apathy [3].

The exact pathophysiology of pCMS is unknown but it is suspected that functional and/or anatomical interruption of the reciprocal cerebello-cerebral pathway plays a vital role [4–6]. Damage to this pathway may lead to diaschisis: a sudden decrease in input from the dentato-thalamo-cerebral (DTC) tract that results in a temporary loss of function of corresponding parts of the cerebral cortex [7]. Risk factors for pCMS that were significant in multiple studies are midline location of the tumor [8], brainstem invasion [8, 9], the tumor being a large size (> 5 cm in diameter) medulloblastoma [4, 7, 8], and presurgical language impairment (PLI)) [6, 10, 11].

The onset of pCMS is delayed by hours to several days after surgery [4, 9], may last from a few days to several months [5], and the mutism resolves spontaneously [2, 4, 9]. Other symptoms may not normalize completely [4, 7, 10, 12–15]. Long-term neurological symptoms, including persistent ataxia, deficits of language and speech, and intellectual handicaps are reported in children with pCMS symptoms of more than 4 weeks duration after medulloblastoma surgery [6, 9, 10, 16, 17]. Also, more severe long-term neuropsychological deficits were found in children with pCMS 1 year after medulloblastoma surgery compared with a matched medulloblastoma group without pCMS [18].

**Fig. 1:**
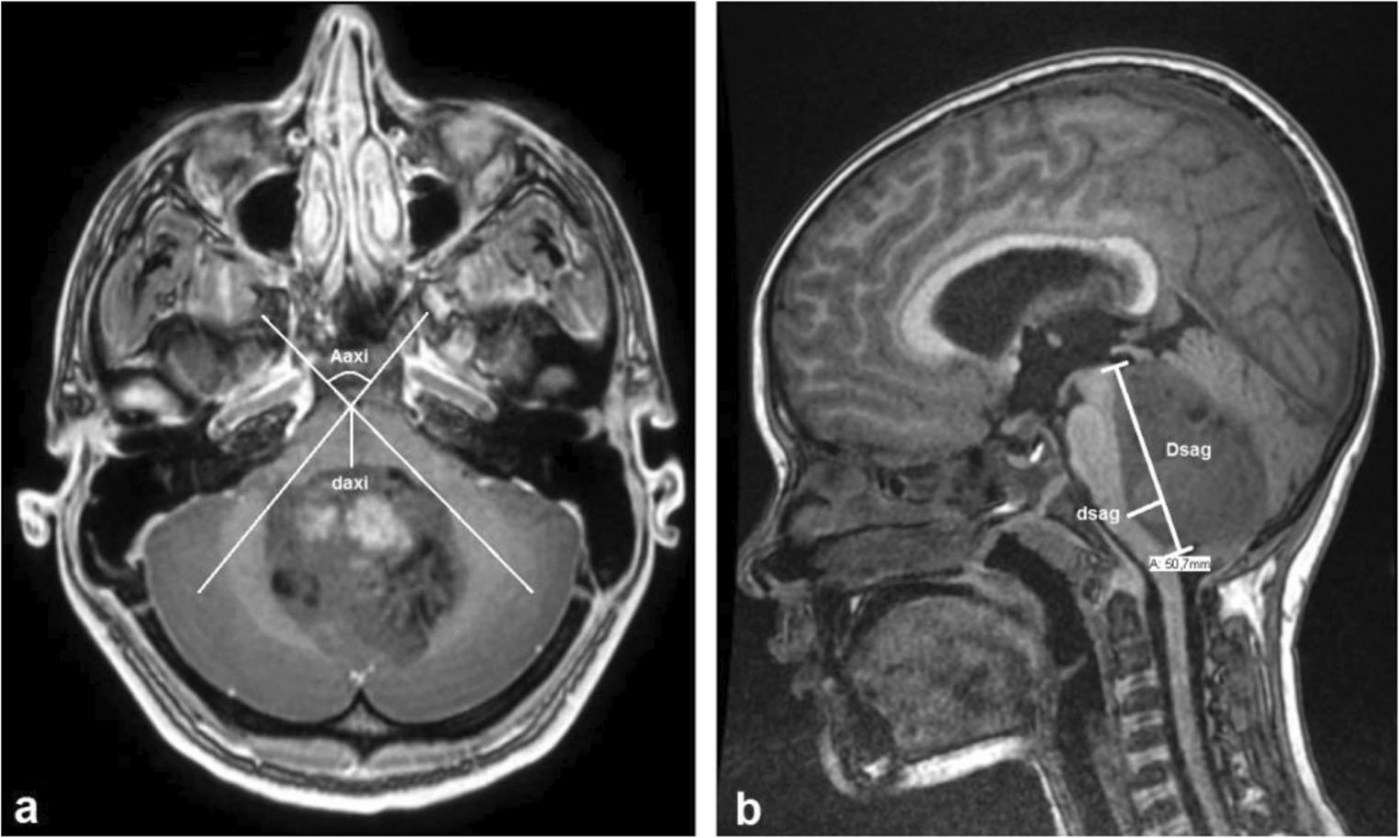
Measurements proposed by Zhang et al. [20]. **a** The point where the lines cross is the bottom of the basilar artery. A(axi) represents the angle between the tumor and the basilar artery. d(axi) represents the distance from the artery to the tumor. **b** D(sag) is the length over which the tumor invades the brainstem. d(sag) represents the depth of invasion

Given the severity of these long-term impairments, prevention of pCMS is crucial. An accurate and easy to use risk model that predicts which patient is at high risk for developing pCMS, and which patient is not, would ameliorate preoperative information for patients and parents and could help to stratify patients for relatively sparing surgical techniques [19]. Liu et al. developed a scoring system based on preoperative MRI to predict the chance of pCMS occurrence [19]. Through a retrospective cohort analysis, they identified five predictors that, when put into a model, yielded the highest accuracy and least number of false negatives: cerebellar hemisphere location of the tumor (preventive factor for pCMS), cerebellar hemisphere invasion, bilateral median cerebellar peduncle invasion and/or compression, dentate nucleus invasion, and age at imaging > 12.4 years. Using this model, they reached in their cohort an accuracy of 88.8%, a sensitivity of 96.2%, and specificity of 85.7%. However, to the best of our knowledge, no studies are published in which their results were validated in other cohorts. Recently, Zhang et al. also described reproducible measurable factors on preoperative MRI that proved to be risk factors for pCMS [20]. In this retrospective case matched study of 46 medulloblastoma patients of which 23 had developed pCMS that they found as reproducible predictors:

1. Compression of cerebellum and brainstem (quantified by the A(axi)/d(axi) ratio, A(axi) being the angle between the tumor and the bottom of the basilar artery and d(axi) being the nearest distance from the bottom of the basilar artery to the tumor (Fig. 1))
2. Compression of the upper brainstem (quantified by A(cor)/d(cor) ratio. A(cor) was defined as the angle between the tumor and the bottom of the third ventricle, d(cor) as the nearest distance from tumor to the bottom of the third ventricle)
3. The distance from the upper to lower point of the brainstem invaded by tumor, multiplied by d(sag), the depth of invasion of the brainstem; Dsag*dsag (Fig. 1)
4. Evan’s index as a measure of obstructive hydrocephalus (ratio between the greatest distance of the frontal horns and the brain parenchyma).

However, they did not as yet apply their risk factors into a predictive model. The primary focus of this paper was to apply the Liu et al. scoring system to the children in our cohort and evaluate the reproducibility of their results to predict pCMS after cerebellar tumor surgery [19]. In this cohort, we also evaluated the validity of the measurements reported by Zhang et al. [20] and aimed to ameliorate the prediction model, when applicable.

## Methods

### Study population

We included in this retrospective study all 2–18 years old children who underwent fossa posterior tumor surgery in our hospital between 2004 and 2018. All children with a posterior fossa tumor have a routine postoperative follow-up paying particular attention to signs and symptoms of pCMS by means of neurological evaluations at regular intervals. Patients with missing preoperative MRI-scans or age younger than 2 years were excluded. Language development is limited in this young age group, making an accurate diagnosis of mutism as a part of pCMS difficult. Children were attributed to either the group that developed pCMS or the non-pCMS group. Information on age at surgery, gender, and occurrence and duration of pCMS was collected from the electronic patient system. pCMS was diagnosed according to the definition based on the Iceland Delphi results as described by Gudrunardottir et al. [3].

### Image analysis

**Fig. 2:**
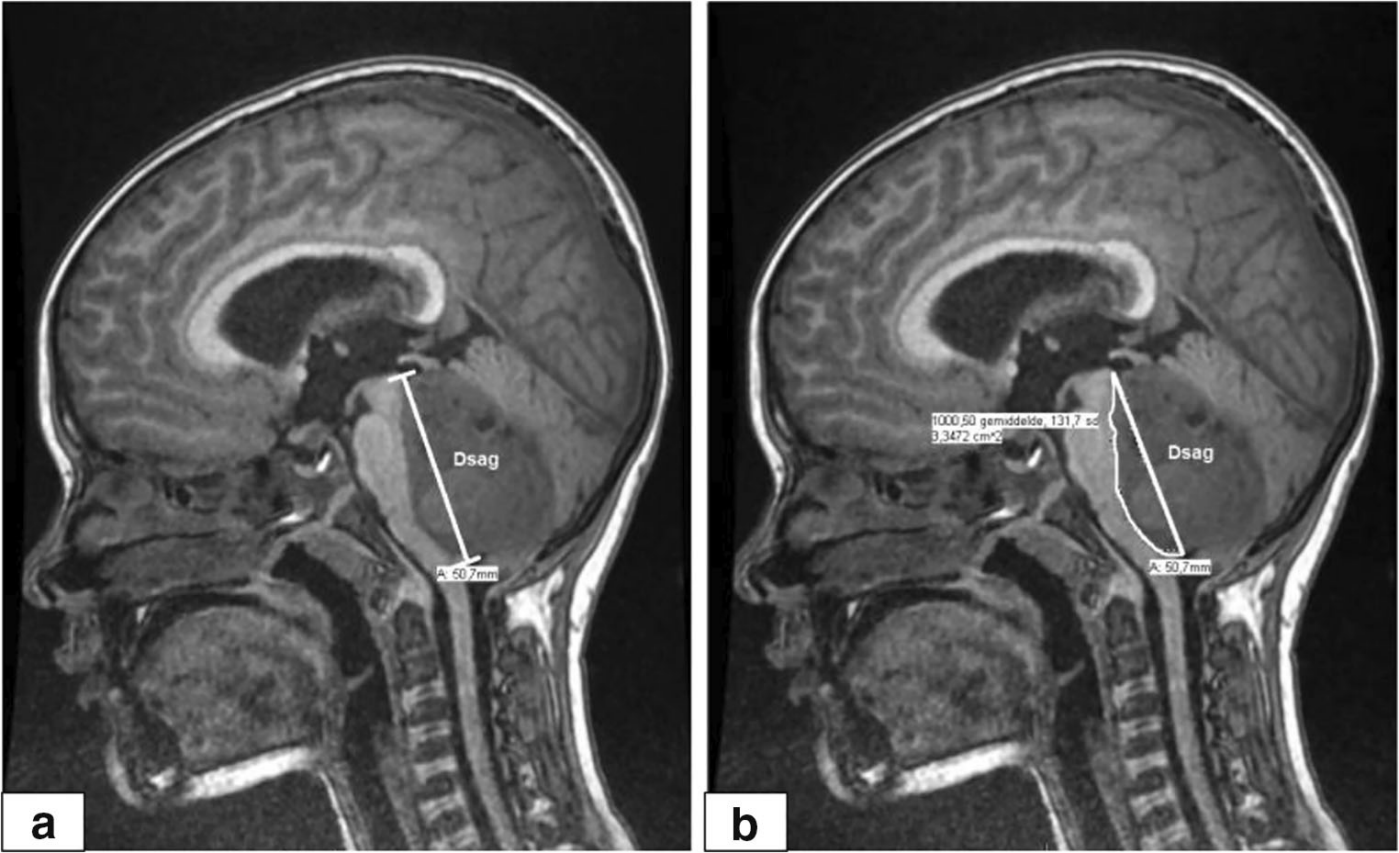
Example of measurement of area: A(sagittal): using the D(sagittal) line in the midline as reference (**a**), the outlines of the tumor that invaded and/or compressed the brainstem were traced (**b**). The estimated size of the area of invasion/compression was calculated by the program used to evaluate the MRI-scans

The imaging features measured and scored in this study were carried out according to the methods used by Liu et al. [19] and Zhang et al. [20] (Table 1). For a definition of imaging features, we refer to the table from the publication by Liu et al. [19] In addition, we decided to measure Zhang et al.’s D(sag) and d(sag) irrespective of the tumor compressing or invading the brainstem. In addition to Zhang et al.’s measures, we calculated in a midsagittal section the tumor area compressing and or invading the brainstem by delineating the tumor compressing/invading the brainstem up to the D(sagittal) line indicating this measure as area sagittal: A(sagittal) (Fig. 2). The MRI-scans were assessed by a trained medical (master) student (BD). Following the initial assessment, an experienced pediatric neurologist also reviewed the scans (CC-B). Results were discussed and adjusted if deemed necessary.

**Table 1:** Definitions of measurements by Zhang et al. [20] and the measurement the area of tumor invasion and/or compression, which were used in our study

Measurement	Unit	Description
A(axial)	Degrees (°)	Angle between bottom of the basilar artery and the tumor.
d(axial)	Centimeters	Nearest distance from basilar artery to tumor.
D(sagittal)	Centimeters	Distance from the upper point to the lower point of the brainstem invaded by the tumor.
d(sagittal)	Centimeters	Depth of the invasion and/or compression of the brainstem by the tumor.
A(sagittal)	Square centimeters	Using the D(sagittal) line as reference, the area of invasion and/or compression of the brainstem by the tumor measured by hand. Measured at the level of greatest compression/invasion (Fig. 2).
Evan’s index		The ratio between the maximal diameter of the frontal horns and the inner diameter of the skull.

### Statistical analysis

The study population was characterized by descriptive statistics. The two groups were compared using *T* test, chi-square, and Fisher’s exact test where appropriate. In order to identify possible risk factors for pCMS, odds ratios (OR’s) and 95% confidence intervals were calculated using logistic regression. Valuables that reached statistical significance (*p* < 0.05) in univariate analysis were then used as input in stepwise, backward, and forward multivariable logistic regression analysis. Risk models were developed based on the results from multivariate logistic regression, goodness of fit, and the classification table calculated by SPSS. Risk models were judged by their applicability and usefulness in the clinical setting. Following the method described by Liu et al. [19], risk scores for each predictor were calculated by adjusting the OR for age and gender, then multiplying the logistic regression coefficients by ten. In order to limit the number of possible total risk scores, the risks scores were truncated to the nearest integer divisible by 5 (5, 10, 15 etc.). Correlation analysis and linear regression were used to evaluate a possible connection between variables and the length of pCMS. All analyses were performed using SPSS version 24 for Windows and Mac.

### Ethical approvals

Because of retrospective nature of the study and the fact that all data were collected as part of usual clinical care, ethical approval was not necessary for this study.

## Results

Of the 160 patients that underwent posterior fossa tumor surgery in the given time period, 39 children were excluded because of missing preoperative MRI (*n* = 14) or age younger than 2 years (*n* = 25). Of the 121 patients included in the analysis, 31 children were attributed to the pCMS group (26%) and 90 children in the non-pCMS group. Relevant data are shown in Table 1. No statistical significant difference in age and gender was found between groups.

On preoperative MRI, 70% of the tumors were located in the midline (vermis and fourth ventricle) and 25% were located in the cerebellar hemispheres. Based on MRI characteristics, the tumor was preoperatively suspected to be a medulloblastoma (MB) in 37%, pilocytic astrocytoma (PA) in 33%, and ependymoma (Ep) in 21% of children. In contrast the final histopathological diagnosis was MB in 47% PA in 43% and Ep in 6% of children (Table 2).

**Table 2:** Distribution of relevant variables in the total cohort, the pCMS and non-pCMS group, with crude odds ratios and *p* values

Patients	Total (*n* = 121)		pCMS (*n* = 31)		Non pCMS (*n* = 90)			
	*n*	%	n	%	*N*	%	OR	*p* value
Age							0.94	0.183
Mean ± SD	9.1 ± 4.6		8.2 ± 4.3		9.5 ± 4.6			
Gender							0.44	0.085
Male	78	64	24	77	54	60		
Female	43	36	7	23	36	40		
Preoperative MRI diagnosis								
Medulloblastoma	45	37	20	65	25	28	4.73	*< 0.001*
Pilocytic astrocytoma	40	33	2	7	38	42	0.09	*0.002*
Ependymoma	25	21	9	29	16	18	1.89	0.186
Other	7	6	0	0	7	8		
Histopathology								
Medulloblastoma	57	47	26	84	31	34	9.89	*< 0.001*
Pilocytic astrocytoma	52	43	4	13	48	53	0.13	*< 0.001*
Ependymoma	7	6	1	3	6	7	0.47	0.489
Other	5	4	0	0	5	6		
Tumor location on MRI								
Vermis	72	59	29	93	43	48	15.85	*< 0.001*
Cerebellar hemisphere	30	25	0	0	30	33		
Fourth ventricle	12	10	2	7	10	11	0.55	0.460
Other	7	6	0	0	7	8		

Values in italics: *p* < 0.05, *n* = number of patients, *pCMS* = postoperative cerebellar mutism syndrome, *OR* = odds ratio

### Risk factors for pCMS

In our cohort, seven variables were found to be significant risk factors for pCMS in univariate logistic regression (Tables 2 and 3): preoperative radiological diagnosis of MB (OR 4.73), histopathological diagnosis of MB (OR 9.9), tumor location on MRI in the vermis (OR 15.85), tumor invasion on MRI into the brainstem (OR 32.79), tumor invasion on MRI into the fourth ventricle (OR 26.25), and tumor invasion on MRI into the middle (MCP) (OR 16.55), and superior cerebellar peduncle (SCP) (OR 11.08). PA, either by radiological diagnosis (OR 0.09) or histopathological diagnosis (OR 0.13), reduced pCMS risk. Also, tumor location in the cerebellar hemisphere showed a protective effect: of the 30 patients with a tumor in this location, none developed pCMS. Compression of a cerebellar hemisphere also slightly reduced pCMS risk (OR 0.25).

**Table 3:** Distribution of MRI features in the pCMS and non-pCMS group, with corresponding crude odds ratios and 95% confidence intervals

Patients	Total (*n* = 121)		pCMS (*n* = 31)		Non pCMS (*n* = 90)				
	*n*	%	*n*	%	*n*	%	OR	*p* value	95% CI
Vermis invasion	99	81.8	31	100	68	75.6			
Vermis compression	116	95.9	31	100	85	94.4			
Brainstem invasion	73	60.3	30	96.8	43	47.8	32.79	*0.001*	4.286–250.883
Brainstem compression	95	78.5	25	80.6	70	77.8	1.19	0.738	0.429–3.302
4th ventricle invasion	78	64.5	30	96.8	48	53.3	26.25	*0.002*	3.431–200.861
4th ventricle compression	113	93.4	31	100	82	91.1			
CH invasion	68	56.2	13	41.9	55	61.1	0.46	0.066	0.200–1.054
Left	44	36.4	8	25.8	36	40	0.52	0.160	0.210–1.294
Right	33	27.3	8	25.8	25	27.8	0.90	0.832	0.358–2.286
Both sides	9	7.4	3	9.7	6	6.7	1.50	0.584	0.352–6.397
CH compression	55	45.5	7	22.6	48	53.3	0.25	*0.004*	0.100–0.652
Left	40	33.1	6	19.4	34	37.8	0.39	0.066	0.147–1.061
Right	40	33.1	5	16.1	35	38.9	0.30	*0.025*	0.106–0.861
Both sides	25	20.7	4	12.9	21	23.3	0.49	0.223	0.153–1.550
MCP invasion	88	72.7	30	96.8	58	64.4	16.55	*0.007*	2.155–127.111
Left	59	48.8	24	77.4	35	38.9	5.39	*< 0.001*	2.099–13.828
Right	66	54.5	30	96.8	36	40	45	*< 0.001*	5.872–344.870
Both sides	37	30.6	24	77.4	13	14.4	20.31	*< 0.001*	7.274–56.699
MCP compression	78	64.5	24	77.4	54	60	2.28	0.085	0.891–5.861
Left	52	43	19	61.3	33	36.7	2.73	*0.019*	1.180–6.337
Right	50	41.3	22	71	28	31.1	5.41	*< 0.001*	2.212–13.244
Both sides	24	19.8	17	54.8	7	7.8	14.40	*< 0.001*	5.055–41.006
SCP invasion	31	26.1	19	63.3	12	13.5	11.08	*< 0.001*	4.244–28.944
Left	22	18.5	13	43.3	9	10.1	6.80	*< 0.001*	2.505–18.443
Right	18	15.1	13	43.3	5	5.6	12.85	*0.001*	4.045–40.803
Both sides	9	7.6	7	23.3	2	2.2	13.24	*0.002*	2.575–68.065
SCP compression	61	50.4	18	58.1	43	47.8	1.51	0.325	0.663–3.452
Left	48	39.7	16	51.6	32	35.6	1.93	0.118	0.846–4.417
Right	36	29.8	14	45.2	22	24.4	2.54	*0.032*	1.082–5.987
Both sides	23	19	12	38.7	11	12.2	4.54	*0.002*	1.738–11.837

*n* = number of patients, *CH* = cerebellar hemisphere, *MCP* = middle cerebellar peduncle, *SCP* = superior cerebellar peduncle, *pCMS* = postoperative cerebellar mutism syndrome, *OR* = odds ratio. Values in italics: *p* < 0.05

### The Liu et al. model

**Fig. 3:**
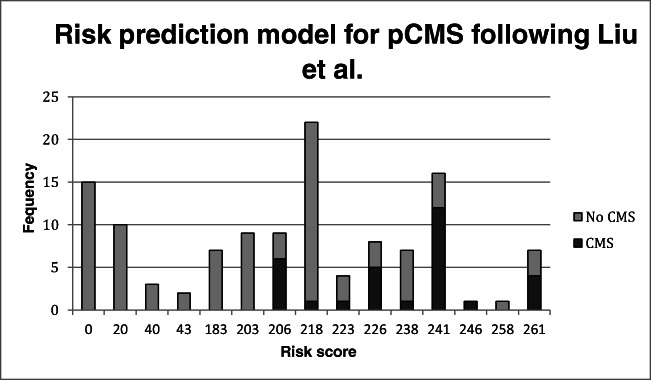
Distribution of risk scores for pCMS in our cohort using the prediction model for pCMS developed by Liu et al. [19]. Cut off point for high risk to develop pCMS in their model is 238 points

We used the risk prediction model developed by Liu et al. [19] in our cohort to test the model accuracy. The distribution of the risk scores is represented in Fig. 3. Using their proposed cut-off point of 238 for a high risk of pCMS, we found that in our cohort the Liu et al. model reached an accuracy of 78% (94/121 patients correctly predicted), a sensitivity of 58% (18/31), and a specificity of 84% (76/91).

### Measurements of Zhang et al.

When assessing the measurements proposed by Zhang et al. [20], we found the following results (Table 4). Evan’s index gave a significant OR, although the effect size was small (OR 1.07). In axial MRI images, both A(axial) and d(axial) turned out to be insignificant risk factors for pCMS (OR of 1.03 and 0.25, respectively). The ratio of A(axial) divided by d(axial) was also not a significant risk factor (OR 1.01, *p* value 0.095). In the sagittal plane, D(sagittal) and d(sagittal) were significant risk factors for CMS (OR 2.95 and 11.06 respectively), as was the product of D(sagittal) and d(sagittal) (OR 2.07). A(sagittal) also proved to be a significant risk factor with an OR of 2.93.

**Table 4:** The mean and standard deviation (SD) of the measurements following Zhang et al. [20] and A(sagittal) in the pCMS and non-pCMS group, with corresponding odds ratios (OR) and 95% confidence interval (CI)

Patients	Total (*n* = 121)	pCMS (*n* = 31)	Non pCMS (*n* = 90)			
	Mean (±SD)	Mean (±SD)	Mean (±SD)	OR	*p* value	95% CI
Evan’s index	0.29 (0.10)	0.33 (0.07)	0.28 (0.10)	1.07	*0.007*	1.020–1.128
∠A axial	69.9 (22.6)	80.5 (16.3)	66.5 (23.4)	1.03	*0.006*	1.009–1.052
d axial (cm)	1.55 (0.69)	1.22 (0.39)	1.66 (0.73)	0.25	*0.005*	0.098–0.640
Ratio ∠A/d	60.7 (50.6)	75.6 (34.8)	55.8 (54.1)	1.01	0.095	0.999–1.015
D sagittal (cm)	2.91 (1.61)	4.07 (1.05)	2.51 (1.58)	2.49	*< 0.001*	1.596–3.897
d sagittal (cm)	0.44 (0.33)	0.62 (0.26)	0.37 (0.33)	11.06	*0.002*	2.460–49.736
Product of D*d	1.62 (1.44)	2.70 (1.60)	1.25 (1.18)	2.07	*< 0.001*	1.746–4.915
A sagittal (cm^2^)	1.00 (0.92)	1.67 (0.96)	0.77 (0.78)	2.93	*< 0.001*	1.746–4.915

*pCMS* = postoperative cerebellar mutism syndrome. *SD* = standard deviation. Values in italics: *p* < 0.05

### The Rotterdam model

Considering the facts that, in our cohort, the model of Liu et al. [19] had a relatively low model accuracy and sensitivity and that Zhang et al. [20] provided measurements that were significant risk factors of varying effect size and were not as yet implemented into a risk prediction model, we made a new risk prediction model for pCMS combining results from these two studies and our analysis.

Following the method described by Liu et al. [19], all variables that were significant risk factors for pCMS in univariate analysis were used as input in multivariate logistic regression to select predictors for the prediction model. Potential protective variables, such as cerebellar hemisphere tumor location, were also included in prediction models.

**Fig. 4:**
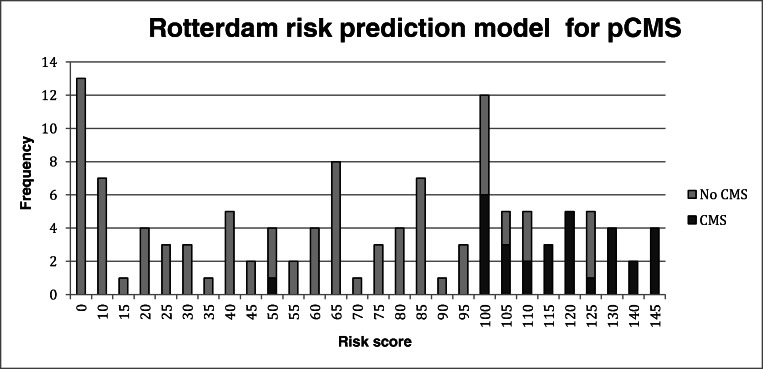
Distribution of risk scores in our cohort using the now newly developed Rotterdam pCMS prediction model. Scores 0–49 represent a low predicted probability, scores 50–99 represent intermediate predicted probability, and scores of 100 and higher represent a high predicted probability of developing pCMS

Predictors used in the optimal model are represented in Table 5. In our cohort, this model reaches an accuracy of 87% (105/121), a sensitivity of 97% (30/31), and a specificity of 84% (75/91). Risk factors that are included are as follows: radiological diagnosis of MB, midline tumor location on preoperative MRI, invasion of the tumor in the middle cerebellar peduncle (MCP: right sided invasion and bilateral invasion were greater risk factors than left sided invasion, Table 3) and invasion of the tumor in the superior cerebellar peduncle (SCP: right sided invasion and bilateral invasion was a greater risk factor than left sided invasion, Table 3). The total calculated risk scores ranged from 0 to 145 (Fig. 4). A higher risk score is associated with an increased predicted risk of pCMS. Using cut-off scores of 50 and 100 splits, the total risk scores into three groups: scores 0–49 representing a low predicted probability, scores 50–99 an intermediate predicted probability, and scores of 100 and higher a high predicted probability of developing pCMS. An easy to use calculation tool in an excel file can be found as supplementary Table S1.

**Table 5:** Predictors for pCMS used in the Rotterdam pCMS prediction model with corresponding adjusted OR, 95% confidence interval (CI) and risk score appointed to the predictor

Predictors	Regression coefficient	Adjusted OR (95% CI)*	Risk score
Radiological diagnosis			
Medulloblastoma	1.655	5.234 (2.106–13.011)	15
Other	–	–	0
Tumor location on MRI			
Midline	1.181	3.26 (2.221–8.312)	10
Cerebellar hemisphere	–	–	–
Brainstem invasion	3.486	32.655 (4.232–251.966)	35
Middle cerebellar peduncle			
Invasion left	1.996	7.362 (2.646–20.485)	20
Invasion right	3.915	50.124 (6.371–394.338)	40
Bilateral invasion and/or compression	3.853	47.120 (12.365–179.561)	40
Invasion superior cerebellar peduncle			
Left	1.281	3.602 (1.009–12.859)	10
Right	1.949	7.022 (1.581–31.197)	20
Bilateral	2.707	14.983 (2.659–84.439)	25
d(sagittal) ≥ 0.58 cm	1.935	6.922 (2.691–17.808)	20

*Adjusted for gender and age. *OR* = odds ratio

## Discussion

Because of the severe long-term neurological sequelae of pCMS, prevention of this syndrome is of utmost importance. We emphasize the need of an easy to use, reliable, and sensitive preoperative risk prediction model to facilitate an intraoperative approach to reduce the occurrence pCMS.

Considering the high model accuracy in the Liu et al. cohort [19], we expected that their model would predict pCMS risk accurately in our cohort as well. However, we found a rather disappointing model accuracy of 78%, a sensitivity of 58%, and a specificity of 84% in our cohort, indicating that the model of Liu et al. is not as accurate as we had hoped. In our cohort, the Liu et al. model did not correctly predict 13 out of 31 pCMS patients (42%). One of our problems with the model of Liu et al. was scoring one of their risk factors, i.e., correct identification of tumor invasion of the dentate nucleus (DN) on a preoperative MRI. Due to compression by often large sized tumors, we could not reliably identify the DN in 55.4% of the patients. We hypothesized that the low sensitivity of the model in our cohort could possibly be explained by our poor assessment of the DN. In order to test if DN tumor invasion had a large impact on model accuracy and sensitivity, we appointed the risk points for DN invasion to every pCMS patient in our cohort. This resulted in a model accuracy of 79% (95/121), a sensitivity of 61% (19/31), and a specificity of 84% (76/91). So theoretically, even if we could have easily identified DN invasion in pCMS patients, the Liu et al. model still would not predict pCMS risk well in our patients as well as in their cohort.

When considering the measurements of Zhang et al. [20], we should mention that we chose not to assess A(cor)and d(cor) and thus not A(cor)/d(cor) ratio because we found it hard to define the bottom of the third ventricle on coronal images. All other measurements of Zhang et al. showed a significant difference between the pCMS and non-pCMS group, except for the ratio between A(axial) and d(axial). We were especially impressed by the measurement illustrating compression/invasion of the dorsal brainstem, i.e., d(sagittal), that reached an impressive odds ratio of 11.06. This measurement made it into our final risk prediction model.

The results from the imaging features mostly match those from Liu et al. [19]. Known risk factors such as brainstem invasion, midline location of the tumor, and tumor type MB were confirmed in this study. A surprising finding was the protective effect of the tumor being a PA. We hypothesized that this effect could be explained by the preferably cerebellar hemisphere location of these tumors, but when analyzing only the midline located tumors, PA retained its protective effect. Considering that this protective factor has not been found in other studies, it is possible that there were more PA in our cohort than in other studies, resulting in skewed results.

Despite the fact that we did not include DN tumor invasion into our analysis, our finding that bilateral more than unilateral SCP compression or invasion are high risk factors to develop pCMS support the hypothesis that pCMS results from damage to the DTC tract. In agreement with Liu et al. [19], we found different odds ratios for invasion into the left, right, and bilateral SCP and therefore different scores were appointed (Table 5).

Also in agreement with Liu et al. [19], we found that tumor compression or invasion into the MCP is a high risk factor for pCMS. The MCP contains the ascending fibers of the cortico-ponto-cerebellar pathway. These fibers originate in the primary motor cortex, enter the ipsilateral pontine nucleus and cross the pons to reach the contralateral cerebellar cortex through the MCP. In turn, the cerebellum returns projections to the motor cortex by way of the DTC tract. This loop of strongly reciprocal fibers is involved in the initiation and execution of (fine) movements, including movements of the mouth and tongue. Damage to the MCP could disrupt this loop, and possibly contribute to onset of pCMS. Until now, focus has always been more on the DTC tract, but the cortico-ponto-cerebellar pathway could play an unrecognized part in pCMS pathophysiology. We found different odds ratios for invasion into the left, right, and bilateral peduncle and therefore different scores were appointed.

The strength of our study is that the risk factors used in our new model are easy to identify on preoperative MRI in daily practice. Of course, we acknowledge that our study has limitations. Images were assessed by two researchers and MRI assessments were done using a standardized assessment form. Secondly, in contrast to Liu et al. [19], we did not use decision tree analysis when creating the risk prediction model. It is possible that variable inclusion into the model would have been different if we had used a decision tree. Finally, the sample size and number of events (*n* = 31) used in this study was relatively small. This may lead to less reliable and skewed statistical results. As an example, some variables such as brainstem invasion show very large odds ratios with a wide confidence interval. We acknowledge this as a limitation to this study. However, given the fact that our results match those of Liu et al. [19], we are confident that our results give a good indication on which preoperative imaging features and variables influence pCMS risk. Ideally, multiple cohorts will be combined in the future, to validate the current prediction models. In earlier studies, PLI was strongly predictive of pCMS [6, 10, 11]. In the present study, we could not insert data in the model on preoperative language function because in our institution children that are admitted with a brain tumor are not routinely assessed neuropsychologically before surgery. Inserting results of presurgical language evaluation as proposed by Bianchi et al. [10] could possibly further ameliorate accuracy and specificity of the present MRI based model.

The tumor being a large sized (> 5 cm diameter) medulloblastoma and midline location are accepted greatest risk factors for developing pCMS [4, 7, 8]. In the past few decades, radical resections seemed to be the norm at least when treating patients with medulloblastoma. Given the hypothesis at that time that especially in children with medulloblastoma gross total resection improved survival, neurosurgeons usually attempt to remove all visible tumor, often at the expense of the DTC tract and other vulnerable cerebellar structures. However, in the last few years, it has become apparent that gross total resection not only increases pCMS risk but also does not improve survival in medulloblastoma compared with near total resection (residue less than 1.5 cm^2^) [21, 22]. For this reason, we have to ask ourselves if a possible minimal increased gain in chance of survival is worth the high risk of developing pCMS and its severe long-term consequences. We strongly advocate a step-wise strategy in intraoperative tumor approach in case of suspicion of MB on preoperative MRI and/or preoperative confirmation. Our study confirms that it is of utmost importance to aim at preserving the middle and superior cerebellar peduncles at least on one side, preferably the right side. Supported by studies that have shown that a complete resection does not improve survival over a subtotal resection with a residue less than 1.5 cm^2^, leaving such a small residue on the peduncle may be acceptable in order to preserve this structure critical in pCMS prevention. Strategies could start by dissecting the side that shows less infiltration on MRI and if indeed easy to dissect without harming the peduncle to proceed with a more radical resection on the contralateral side. If resection is difficult, it would be sensible to leave a small residue on the first side and adapt the extent of resection on the contralateral peduncle to preserve at least one peduncle or even accept to leave small residues on both peduncles.

## Conclusion

We were unable to reproduce the accuracy of the pCMS prediction model as described by Liu et al. [23] in our cohort of children that underwent posterior fossa tumor surgery. We updated the Liu et al. pCMS prediction model to a new, easy to use in daily practice pCMS risk prediction model. This model reached a high accuracy in our cohort. After prospective validation of this pCMS risk prediction model, it could assist neurosurgeons in determining their surgical tactics in order to prevent pCMS if possible and help prepare high risk patients and their parents for this severe complication.

## Electronic supplementary material

ESM 1 (XLSX 673 kb)
